# Education, material condition and physical functioning trajectories in middle-aged and older adults in Central and Eastern Europe: a cross-country comparison

**DOI:** 10.1136/jech-2015-206548

**Published:** 2016-05-18

**Authors:** Yaoyue Hu, Hynek Pikhart, Andrzej Pająk, Růžena Kubínová, Sofia Malyutina, Agnieszka Besala, Anne Peasey, Michael Marmot, Martin Bobak

**Affiliations:** 1 Department of Epidemiology and Public Health, University College London, London, UK; 2 Department of Epidemiology and Population Studies, Faculty of Health Sciences, Jagiellonian University Medical College, Krakow, Poland; 3 National Institute of Public Health, Prague, Czech Republic; 4 Institute of Internal and Preventive Medicine, Novosibirsk, Russia; 5 Novosibirsk State Medical University, Novosibirsk, Russia

**Keywords:** Epidemiology of ageing, Health inequalities, PHYSICAL FUNCTION, Cohort studies, LONGITUDINAL STUDIES

## Abstract

**Background:**

Two competing hypotheses, cumulative advantage/disadvantage and age-as-leveller, have been proposed to explain the contradictory findings on socioeconomic differences in health over the lifespan. To test these hypotheses, this investigation examined the influence of educational attainment and material condition on individual trajectories of physical functioning (PF) in unexplored ageing populations in Central and Eastern Europe.

**Methods:**

28 783 men and women aged 45–69 years selected from populations in seven Czech towns, Krakow (Poland) and Novosibirsk (Russia). PF was measured by the Physical Functioning Subscale (PF-10) of the Short-Form-36 questionnaire (SF-36) at baseline and three subsequent occasions. The highest educational attainment was self-reported at baseline, and material condition was captured by the sum score of 12 household amenities and assets.

**Results:**

In all cohorts, participants with a university degree had the highest PF-10 score at baseline and slowest rate of decline in the score during follow-up, while the lowest baseline scores and fastest decline rate were found in participants with less than secondary education in all cohorts and in Russians with secondary education. Similar disparities in the baseline PF-10 score and decline rate were observed across tertiles of material condition, but differences in decline rates across the three tertiles among Czechs or between the lower two tertiles among Russians were not statistically significant.

**Conclusions:**

Disparities in PF by educational attainment and material condition among middle-aged and older adults in Central and Eastern Europe existed at baseline and widened during ∼10 years of follow-up, supporting the cumulative advantage/disadvantage hypothesis.

## Introduction

Physical functioning (PF) is a key domain of healthy ageing, and it is strongly related to older adults’ quality of life.^1^ ^2^ Decline in PF with age is a consequence of physiological changes and onset of diseases, and it can be modified by medical care, socioeconomic, environmental, behavioural and psychosocial factors.^3^ ^4^

Socioeconomic position (SEP) is a well-established determinant of health, in western countries and in Central and Eastern Europe (CEE).^5–7^ Studies on socioeconomic differences in health over the lifespan led to two competing hypotheses: the cumulative advantage/disadvantage (CAD) hypothesis and age-as-leveller (AAL) hypothesis.^8–11^ The CAD hypothesis suggests that socioeconomic disparities in health widen with increasing age, reflecting an accumulation of disadvantages in economic, social, psychological and behavioural resources over the life course.^9–11^ The AAL hypothesis predicts diminishing socioeconomic gaps in health in late life.^8^ ^10^ This convergence may be due to a biological ceiling (as older adults from a different SEP background may become universally fragile with age) or selective mortality (whereby unhealthy persons from lower SEP groups die before reaching an old age while healthy persons survive).^8^ ^10^ ^11^

Several prospective studies have supported the CAD hypothesis with regard to the associations of SEP with walking speed,^12^ functional limitations,^9^ ^13^ disability^13^ ^14^ and physical health.^15^ However, there is also some support for the AAL hypothesis. Taylor^16^ found no association of education and income with either onset or rate of change in severity of disability in Americans aged 85 years and over. A similar lack of relationship between a composite indicator of SEP (education, income and occupation) and rate of decline in grip strength over a 13-year follow-up was shown in Germans aged 70 years and older,^17^ while growing gaps in PF by education and income over a 9-year follow-up were found among Dutch people aged 55–70 years but not among those aged 70–85 years.^18^

To the best of our knowledge, no previous study has tested these two hypotheses by examining socioeconomic disparities in PF changes with age in CEE. In this cross-country comparison, we investigated the relationships of educational attainment and material condition with individual PF trajectories over ∼10 years of follow-up in three cohorts of middle-aged and older persons in the Czech Republic, Poland and Russia.

## Methods

### Data

We used data from the Health, Alcohol and Psychosocial factors In Eastern Europe (HAPIEE) study.^19^ The baseline survey in 2002–2005 examined 28 783 men and women aged 45–69 years randomly selected from population registers in seven middle-sized towns in the Czech Republic (Havířov/Karviná, Jihlava, Ústí nad Labem, Liberec, Hradec Králové and Kroměřiz) and Krakow (Poland), and from electoral lists in Novosibirsk (Russia), stratified by sex and 5-year age groups. Czech and Polish participants completed a structured questionnaire during a home visit, and then were invited to a short medical examination in a clinic. Russian participants completed both in a clinic. The questionnaire was translated into local languages and back-translated into English to ensure accuracy and cross-cultural comparability and then piloted in a separate sample.^20^ Participants were re-examined in 2006–2008 using a Computer-Assisted Personal Interview, and further followed up in 2009 (PQ2009) and 2012 (PQ2012) using postal questionnaires. The HAPIEE study was approved by ethics committees at University College London and all local centres. All participants gave informed consent.

### Physical functioning

PF was measured by the physical functioning subscale (PF-10) of the Short-Form-36 questionnaire (SF-36) on each occasion.^21^ The PF-10 evaluates limitations in 10 items regarding vigorous activities (eg, doing strenuous sports), moderate activities (eg, moving a table), lifting/carrying a bag of groceries, walking, climbing stairs, bending, kneeling or stooping, and bathing and dressing. Participants rated themselves as being ‘limited a lot’, ‘limited a little’ or ‘not limited at all’ to each item. A summary score (0–100) was calculated, with a higher score indicating better PF.^22^

### Highest educational attainment and material condition

Highest educational attainment and material condition were self-reported at baseline. The highest educational attainment was categorised into less than secondary education (incomplete primary education or no formal education, primary education, vocational education and apprenticeship), secondary education and university degree. Material condition was determined by 12 household amenities and assets, including mobile phone, telephone, microwave, dishwasher, washing machine, freezer, video recorder, video camera, television, satellite/cable TV, car and cottage. These items were selected to be comparable across the three countries. The sum of items possessed by participants was calculated and categorised by country-specific tertiles.

### Covariates

Besides age at baseline, several baseline characteristics were included as covariates. Marital status was dichotomised into married/cohabiting or not. Participants reported whether they had been diagnosed or hospitalised for spine or joint problems in the past year before baseline. Body mass index (BMI, kg/m^2^) was calculated using objectively measured height and weight. Smoking status was coded as never, former and current smoking. Drinking pattern was derived from a graduated frequency questionnaire: >4 drinks/day (>2 drinks/day for women) was used to classify heavy versus light-to-moderate drinking; these groups were further categorised into regular versus irregular drinking by drinking frequency ≥1/week (≥1/month for heavy female drinkers).

### Statistical analysis

Missing data were handled by multiple imputation by chained equations (MICE), a statistical technique to replace missing data based on available observed data taking into account the uncertainty of missing data.^23^ ^24^ MICE was selected because of its flexibility when dealing with a large data set and different types of variables.^25^ A total of 18 583 participants of the total sample (65%) had missing PF-10 scores from at least one measurement occasion; 11 707 of these 18 583 participants (63%) had information on the scores from two or more occasions. Given the strong correlations between the repeatedly measured PF-10 scores (correlation coefficients 0.54–0.73), inclusion of PF-10 scores from other occasions into the imputation models improved the effectiveness and reliability of imputed scores. During follow-up, 791 Czechs (9.0%), 1109 Russians (11.9%) and 830 Poles (7.8%) died. Compared with survivors, deceased participants disproportionally contained those from lower SEP groups and those with poor baseline PF and faster PF decline (selective mortality). Taking this into account, missing PF-10 scores due to death were imputed. Seventy imputed data sets were generated in a wide format using Stata V.12 (StataCorp, 2013) as the number of imputed data sets is recommended to be equal to or greater than the proportion of cases with missing values on at least one study variable.^23^ Missing follow-up years due to non-response to follow-up were replaced by random numbers generated under normal distribution of observed follow-up years.

Individual trajectories of the PF-10 score during follow-up in the multiply imputed data sets were estimated by latent growth curve modelling in M*plus 6* (Muthén & Muthén, 1998–2011). The model captures interindividual variations in intraindividual growth trajectories.^26^ Visual inspection indicated a linear decline in the PF-10 score over the 10-year follow-up in all cohorts; thus, a linear latent growth curve model was used. Two growth parameters describe the linear PF-10 trajectories: initial status of the score at baseline and rate of change in the score per year of follow-up (slope). Taking into account the longitudinal nature of our data, residuals of adjacent assessment occasions were correlated. In addition, since measurement errors between the first two assessments (face-to-face interview) and latter two assessments (postal questionnaires) were likely to be different, we constrained the variances of the residuals in the first two assessments to be equal, and the same was carried out for the latter two assessments. All models were estimated by maximum likelihood estimation with robust SEs due to the non-normality of the PF-10 scores, and performed separately by cohort.

Both growth parameters were regressed on baseline age (centred on 58 years) and quadratic baseline age to test the birth cohort effect. Quadratic age was not statistically significant on either growth parameter. Subsequently, two models were estimated: (1) adjusted for sex and baseline age (model 1); (2) adjusted for sex, baseline age, marital status, history of spine/joint problems, BMI, drinking pattern and smoking status (model 2). Furthermore, we tested the interactions of baseline age with educational attainment and material condition on the slope of PF-10 decline separately,^10^ but none were statistically significant. Additional sensitivity analysis was performed among complete cases (participants who survived until PQ2012 and had no missing data on all variables used in the model).

The age trend of PF-10 score at baseline and its decline during follow-up by educational attainment and material condition were illustrated simultaneously in ageing-vector graphs.^11^ ^27^ In the ageing-vector graphs, the starting point of the arrow represents the predicted initial status of PF-10 score at baseline. The arrow indicates the direction of the change in PF-10 score during follow-up. All ageing-vector graphs were based on results from model 2 and produced in Stata.

## Results

Table 1 summarises the average sample characteristics of the multiply imputed data sets. The follow-up years in the three cohorts varied from 6.7 to 10.7 years (mean: 8.6). On average, the Czechs had higher PF-10 scores at baseline and underwent a much smaller decline in the score over time (baseline vs PQ2012: 6.0 points) than the Russians (17.9 points) and Poles (14.7 points). The distribution of educational attainment was similar in the Russians and Poles, while more Czechs had less than secondary education, reflecting the smaller size of Czech towns and the tradition of formal vocational training. The Czechs possessed the highest number of household amenities and assets, followed by the Poles, and the Russians owned the least. The PF-10 scores at follow-up were lower in the imputed data sets than in the observed data set (see online supplementary table S1) because healthier participants were more likely to participate in the follow-up surveys.

**Table 1:** Average sample characteristics in the imputed data sets, stratified by country

		Country	
	Czech Republic	Russia	Poland
Total	8773	9301	10 709
Age (years, %)			
45–49	16.9	17.0	18.5
50–54	19.8	19.5	20.7
55–59	19.1	21.6	21.0
60–64	23.0	19.0	19.9
65–69	21.2	22.9	19.9
Sex (%)			
Men	46.4	45.6	48.7
Women	53.6	54.4	51.3
PF-10 score			
Baseline			
Mean	83.4	81.8	80.4
SD	18.9	20.4	21.4
Re-examination			
Mean	81.9	79.7	74.0
SD	18.2	22.4	21.3
PQ2009			
Mean	78.6	68.8	70.9
SD	23.0	27.6	27.0
PQ2012			
Mean	77.4	63.9	65.7
SD	24.2	29.6	27.5
Education (%)			
<Secondary education	49.8	36.9	32.7
Secondary education	36.5	34.2	38.7
University	13.7	28.9	28.5
Material condition			
Mean	6.8	5.7	6.4
SD	2.3	2.1	2.2
1st tertile			
Mean	4.7	3.9	4.0
SD	1.2	1.0	1.1
%	43.6	49.3	35.4
2nd tertile			
Mean	7.5	6.4	6.8
SD	0.5	0.5	0.8
%	31.5	29.4	40.6
3rd tertile			
Mean	9.8	8.8	9.3
SD	1.0	0.9	1.0
%	24.9	21.4	24.0
Marital status (%)			
Married/cohabiting	75.7	72.4	76.3
Single/divorced/widowed	24.3	27.6	23.7
Spine/joint problems (%)			
No	44.0	34.6	30.6
Yes, never hospitalised	43.0	56.0	61.1
Yes, hospitalised	13.0	9.4	8.4
BMI (%)			
<25.0	24.2	27.5	25.5
25.0–29.9	44.0	37.5	43.2
≥30.0	31.8	35.1	31.3
Drinking pattern (%)			
Non-drinking	13.0	15.8	34.4
Irregular light-to-moderate	31.8	42.8	31.6
Regular light-to-moderate	19.7	10.3	14.7
Irregular heavy	26.2	21.4	15.8
Regular heavy	9.3	9.7	3.4
Smoking (%)			
Never	43.9	58.1	39.6
Former	29.5	13.6	28.2
Current	26.6	28.2	32.2

BMI, body mass index; PF-10, physical functioning subscale (10 item).

10.1136/jech-2015-206548.supp2 Supplementary data

Table 2 reports the associations of educational attainment with baseline level and longitudinal decline in PF. After controlling for sex and baseline age (model 1), gradients in both baseline PF (initial status, representing a cross-sectional relationship) and the rate of PF decline (slope, representing a longitudinal relationship) across educational groups were observed among the Czechs and Poles. Among the Russians, there was no difference in either the initial status or slope between the two lower educational groups. Tertiary educated Russians, however, had a higher baseline score and experienced a slower PF decline compared to those with the lowest education. Further adjustment for marital status, history of spine/joint problems and behavioural factors (model 2) did not change the patterns, but the differences in the slope attenuated by some 20% in the Czechs, 17% in the Russians and 12% in the Poles and the variances of initial status and slope were somewhat reduced.

**Table 2:** Initial status at baseline and slope of decline (per 1 year) in the PF-10 scores by educational attainment, stratified by country

	Model 1†				Model 2‡			
	Initial status		Slope		Initial status		Slope	
	Coefficient (95% CI)	p Value	Coefficient (95% CI)	p Value	Coefficient (95% CI)	p Value	Coefficient (95% CI)	p Value
Czech Republic								
Mean§	83.12 (82.42 to 83.82)	<0.01	−0.89 (−1.03 to −0.76)	<0.01	85.10 (83.79 to 86.41)	<0.01	−1.21 (−1.47 to −0.96)	<0.01
Variance§	259.42 (240.12 to 278.72)	<0.01	0.53 (0.05 to 1.01)	0.03	193.25 (174.89 to 211.60)	<0.01	0.48 (0.01 to 0.96)	0.04
Baseline age (year)¶	−0.60 (−0.65 to −0.55)	<0.01	−0.04 (−0.05 to −0.03)	<0.01	−0.37 (−0.42 to −0.32)	<0.01	−0.04 (−0.05 to −0.03)	<0.01
Female	−3.64 (−4.37 to −2.91)	<0.01	0.15 (0.03 to 0.27)	0.02	−1.96 (−2.69 to −1.23)	<0.01	0.13 (0.00 to 0.26)	0.06
Education								
<Secondary education	Reference		Reference		Reference		Reference	
Secondary education	4.99 (4.17 to 5.81)	<0.01	0.21 (0.07 to 0.34)	<0.01	2.84 (2.09 to 3.59)	<0.01	0.17 (0.03 to 0.31)	0.01
University	7.15 (6.18 to 8.11)	<0.01	0.29 (0.13 to 0.45)	<0.01	3.87 (2.95 to 4.78)	<0.01	0.22 (0.06 to 0.38)	0.01
Russia								
Mean§	87.26 (86.38 to 88.15)	<0.01	−2.27 (−2.52 to −2.03)	<0.01	87.62 (86.02 to 89.22)	<0.01	−2.82 (−3.21 to −2.42)	<0.01
Variance§	167.54 (143.74 to 191.34)	<0.01	1.34 (0.59 to 2.09)	<0.01	138.87 (115.34 to 162.41)	<0.01	1.19 (0.40 to 1.97)	<0.01
Baseline age (year)¶	−0.63 (−0.69 to −0.58)	<0.01	−0.11 (−0.12 to −0.09)	<0.01	−0.53 (−0.59 to −0.48)	<0.01	−0.11 (−0.12 to −0.10)	<0.01
Female	−9.43 (−10.24 to −8.63)	<0.01	−0.04 (−0.24 to 0.16)	0.66	−6.85 (−7.88 to −5.81)	<0.01	−0.06 (−0.30 to 0.19)	0.65
Education								
<Secondary education	Reference		Reference		Reference		Reference	
Secondary education	−0.56 (−1.53 to 0.41)	0.26	0.04 (−0.15 to 0.22)	0.71	−0.46 (−1.39 to 0.47)	0.33	0.04 (−0.15 to 0.22)	0.71
University	3.86 (2.93 to 4.79)	<0.01	0.60 (0.41 to 0.79)	<0.01	3.01 (2.10 to 3.92)	<0.01	0.50 (0.31 to 0.69)	<0.01
Poland								
Mean§	80.92 (80.11 to 81.73)	<0.01	−1.92 (−2.09 to −1.76)	<0.01	85.03 (83.61 to 86.44)	<0.01	−2.30 (−2.60 to −2.01)	<0.01
Variance§	258.72 (231.43 to 286.01)	<0.01	1.13 (0.65 to 1.60)	<0.01	194.94 (169.27 to 220.61)	<0.01	0.99 (0.51 to 1.47)	<0.01
Baseline age (year)¶	−0.75 (−0.80 to −0.69)	<0.01	−0.05 (−0.06 to −0.04)	<0.01	−0.58 (−0.64 to −0.52)	<0.01	−0.05 (−0.06 to −0.04)	<0.01
Female	−6.77 (−7.52 to −6.02)	<0.01	−0.17 (−0.31 to −0.03)	0.02	−4.53 (−5.35 to −3.70)	<0.01	−0.16 (−0.32 to 0.00)	0.06
Education								
<Secondary education	Reference		Reference		Reference		Reference	
Secondary education	2.35 (1.38 to 3.33)	<0.01	0.41 (0.23 to 0.59)	<0.01	1.69 (0.75 to 2.62)	<0.01	0.36 (0.18 to 0.54)	<0.01
University	6.32 (5.35 to 7.29)	<0.01	0.75 (0.56 to 0.94)	<0.01	4.31 (3.36 to 5.25)	<0.01	0.65 (0.45 to 0.85)	<0.01

†Adjusted for baseline age and sex.

‡Adjusted for baseline age, sex, marital status, history of spine/joint problems, BMI, drinking pattern and smoking status.

§Conditional on the covariates adjusted in the model.

¶Centred on 58 years.

BMI, body mass index; PF-10, physical functioning subscale (10 item).

**Figure 1:**
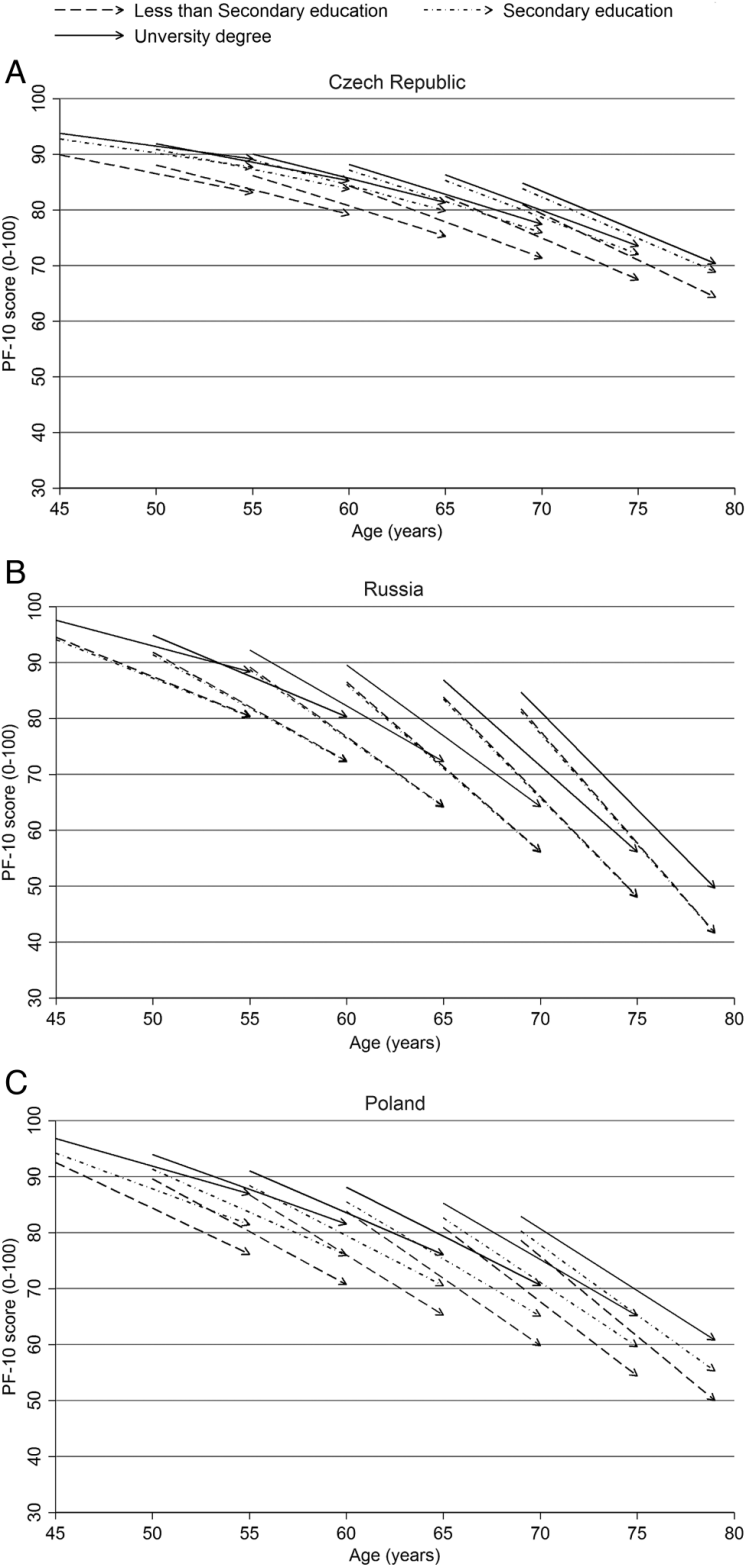
Vector graphs of predicted initial status and slope of PF-10 score during the 10-year follow-up for every fifth one-year birth cohort by educational attainment (model 2) (Czech Republic) (Russia) (Poland). PF-10, physical functioning subscale (10 item).

Figure 1 illustrates the pattern of results using ageing-vector graphs to plot the PF trajectories by educational attainment for six 1-year birth cohorts (age of 45, 50, 55, 60, 65 and 69 at baseline). The differences in PF between the highest and lowest educational categories widened over time in all birth cohorts and in all countries, although the pattern was most pronounced in the Polish and Russian cohorts (p value for interaction between education and cohort on the slope <0.001).

The associations between material condition and the PF trajectories are presented in table 3. In model 1, gradients in the initial status and slope of the PF decline across tertiles of material condition were seen in the Poles and Russians. Among the Czechs, a similar gradient was only found in the initial status but not in the slope. The results remained largely the same after further adjustment in model 2. Among the Russians, the difference in the slope between the lower two tertiles was no longer statistically significant. The additional adjustment accounted for around 16% of reduction of the differences in the slope in the Russians and 10% in the Poles, and the variance of initial status and slope decreased by 20% and 11%, respectively. The differences in baseline PF levels and decline rate by material condition are illustrated in online supplementary figure S1. The PF-10 trajectories across tertiles of material condition were parallel in the Czechs but diverged over the follow-up time among the Russians and Poles (p value for interaction between material condition and cohort on the slope=0.02).

**Table 3:** Initial status at baseline and slope of decline (per 1 year) in the PF-10 scores by material condition, stratified by country

	Model 1†				Model 2‡			
	Initial status		Slope		Initial status		Slope	
	Coefficient (95% CI)	p Value	Coefficient (95% CI)	p Value	Coefficient (95% CI)	p Value	Coefficient (95% CI)	p Value
Czech Republic								
Mean§	83.09 (82.28 to 83.90)	<0.01	−0.83 (−0.98 to −0.67)	<0.01	83.95 (82.56 to 85.34)	<0.01	−1.17 (−1.45 to −0.89)	<0.01
Variance§	264.73 (246.76 to 282.70)	<0.01	0.53 (0.05 to 1.01)	0.03	193.56 (175.26 to 211.86)	<0.01	0.48 (0.02 to 0.95)	0.04
Baseline age (year)¶	−0.54 (−0.60 to −0.49)	<0.01	−0.04 (−0.05 to −0.03)	<0.01	−0.32 (−0.37 to −0.27)	<0.01	−0.04 (−0.05 to −0.03)	<0.01
Female	−3.19 (−3.92 to −2.45)	<0.01	0.15 (0.03 to 0.28)	0.02	−1.84 (−2.56 to −1.11)	<0.01	0.13 (0.00 to 0.26)	0.05
Material condition								
1st tertile	Reference		Reference		Reference		Reference	
2nd tertile	3.36 (2.44 to 4.28)	<0.01	0.09 (−0.05 to 0.22)	0.22	2.96 (2.09 to 3.82)	<0.01	0.06 (−0.08 to 0.20)	0.39
3rd tertile	6.16 (5.23 to 7.09)	<0.01	0.08 (−0.07 to 0.23)	0.30	4.76 (3.89 to 5.63)	<0.01	0.05 (−0.10 to 0.21)	0.49
Russia								
Mean§	85.23 (84.37 to 86.10)	<0.01	−2.29 (−2.53 to −2.04)	<0.01	85.08 (83.46 to 86.69)	<0.01	−2.86 (−3.27 to −2.46)	<0.01
Variance§	165.25 (141.91 to 188.59)	<0.01	1.35 (0.59 to 2.10)	<0.01	136.11 (112.99 to 159.22)	<0.01	1.19 (0.40 to 1.97)	<0.01
Baseline age (year)¶	−0.54 (−0.60 to −0.48)	<0.01	−0.10 (−0.11 to −0.09)	<0.01	−0.46 (−0.52 to −0.40)	<0.01	−0.10 (−0.12 to −0.09)	<0.01
Female	−8.87 (−9.68 to −8.05)	<0.01	−0.02 (−0.22 to 0.19)	0.87	−6.65 (−7.68 to −5.62)	<0.01	−0.06 (−0.30 to 0.18)	0.61
Material condition								
1st tertile	Reference		Reference		Reference		Reference	
2nd tertile	4.68 (3.77 to 5.58)	<0.01	0.22 (0.04 to 0.40)	0.02	4.42 (3.44 to 5.39)	<0.01	0.17 (−0.01 to 0.35)	0.07
3rd tertile	5.91 (4.93 to 6.88)	<0.01	0.57 (0.36 to 0.78)	<0.01	5.52 (3.93 to 7.11)	<0.01	0.48 (0.26 to 0.69)	<0.01
Poland								
Mean§	79.71 (78.82 to 80.60)	<0.01	−1.80 (−1.98 to −1.62)	<0.01	83.02 (81.50 to 84.55)	<0.01	−2.25 (−2.56 to −1.94)	<0.01
Variance§	262.71 (235.90 to 289.52)	<0.01	1.16 (0.69 to 1.64)	<0.01	194.60 (169.39 to 219.81)	<0.01	1.01 (0.53 to 1.49)	<0.01
Baseline age (year)¶	−0.68 (−0.73 to −0.62)	<0.01	−0.05 (−0.06 to −0.04)	<0.01	−0.53 (−0.58 to −0.47)	<0.01	−0.05 (−0.06 to −0.04)	<0.01
Female	−5.94 (−6.71 to −5.17)	<0.01	−0.10 (−0.24 to 0.05)	0.20	−4.22 (−5.04 to −3.40)	<0.01	−0.11 (−0.27 to 0.05)	0.19
Material condition								
1st tertile	Reference		Reference		Reference		Reference	
2nd tertile	4.54 (3.40 to 5.67)	<0.01	0.24 (0.06 to 0.42)	<0.01	3.99 (3.00 to 4.99)	<0.01	0.21 (0.03 to 0.39)	0.02
3rd tertile	6.88 (5.80 to 7.95)	<0.01	0.48 (0.28 to 0.68)	<0.01	5.88 (4.82 to 6.94)	<0.01	0.44 (0.24 to 0.65)	<0.01

†Adjusted for baseline age and sex.

‡Adjusted for baseline age, sex, marital status, history of spine/joint problems, BMI, drinking pattern and smoking status.

¶Centred on 58 years.

§Conditional on the covariates adjusted in the model.

BMI, body mass index; PF-10, physical functioning subscale (10 item).

Educational attainment and material condition are intercorrelated.^28^ ^29^ Since Spearman's rank correlations between educational categories and tertiles of material condition were low in all cohorts (Czechs: 0.29; Russians: 0.21; Poles: 0.32), we additionally estimated both SEP indicators simultaneously. The pattern of results remained similar (see online supplementary table S2), and the effects of both indicators on the initial status and slope attenuated in all cohorts. The difference in the PF decline rate was no longer statistically significant between the lower two tertiles of material condition among the Poles. In the complete-case sensitivity analysis, the pattern of the results remained similar, although the socioeconomic differences in baseline PF and the rate of PF decline over time were less pronounced (results not shown).

## Discussion

In this investigation of social disparities in PF trajectories in three CEE cohorts over ∼10 years of follow-up, we found support for the CAD hypothesis. In general, higher SEP was associated with better PF at baseline and a slower PF decline during follow-up. The results were generally consistent across cohorts and in analyses of both educational attainment and material condition.

This study has several limitations. First, PF was assessed by self-report, which could be influenced by factors such as culture, social role, social desirability and cognitive ability.^30^ ^31^ Since these factors may be associated with SEP, the magnitude of social differences in PF may have been misclassified. Second, both educational attainment and material condition were measured at baseline. Reverse causality is more of an issue for material condition than education as the highest educational attainment is usually fixed in early adulthood.^28^ ^29^ However, middle-aged and older adults with poor PF may be forced to exit from the labour market at an early age, affecting their accumulation of material assets. To some extent, this can also affect the covariates measured at baseline which were treated as time invariant in the analyses. We could not study how the change in material condition was associated with PF trajectories, or how the change in behavioural and other factors was related to educational attainment, material condition and PF trajectories. However, since the material condition was assessed by accumulation of household amenities and assets, they may be more likely to reflect long-term status rather than short-term income, especially given the age of the participants. Finally, the proportion of participants with missing data on at least one study variable is relatively large in this study. The missing data imputation procedure assumes that missingness does not depend on unobserved data. This may not be entirely correct because participants with a lower PF tended to be less likely to respond to follow-up. In this case, the imputed PF-10 scores may be higher than the ‘true’ scores, resulting in an underestimated PF decline and underestimated socioeconomic disparities in the decline rates. Similarly, as in an earlier report,^13^ we imputed the missing PF-10 scores due to death on account of selective mortality. The possibly faster PF drop in deceased participants was taken into account by adding mortality indicators into the imputation. We imputed our data in a wide format that does not reflect the multilevel nature of the data, and this may result in underestimated SEs;^32^ however, results of complete-case sensitivity analyses were consistent with the findings from the imputed data. Nevertheless, socioeconomic disparities in the PF trajectories therefore may have been underestimated.

On the other hand, our study has several important strengths. It covers previously unexplored populations with different social histories and relatively poor health status and low life expectancy, compared to western countries.^33^ ^34^ The measurement of PF was based on a widely used instrument, which has been validated in a number of countries including those in this study.^21^ The repeated measure data on PF enabled us to examine the longitudinal PF trajectories by socioeconomic indicators as people grew older. In addition, PF, one of the most critical aspect of healthy ageing, may be of particular importance of public health in CEE as a consequence of high rates of non-communicable diseases and rapid population ageing in this region.^35^ Our findings suggest that continuous efforts should be injected to tackle the socioeconomic inequality in PF among older Central and Eastern Europeans, with particular focus on the oldest of the old. We studied two different domains of SEP, education and wealth, which were designed to be comparable across the three cohorts. The indicator of wealth in this study, household amenities and assets, especially relevant to the older adults, can reflect an accumulation of materials over the life-course and is less influenced by retirement and subsequent change in earning.^29^ The multicentre design optimised the cross-country comparability of our findings. The similarity of findings from different urban ageing populations in our study is consistent with the widely observed social gradient in health^5–7^ and provides support for the CAD hypothesis.

The results are plausible. Education is associated with employment opportunities and wealth, access to information and ability to process information, cognitive ability, ability to develop and change behaviours, and control of life,^28^ ^36–38^ all of which can mediate its association with functional status. Household amenities and assets are linked with access to healthcare, nutrition, housing, transport and other opportunities and resources.^28^ ^37^ ^38^ Therefore, education and material conditions provide plausible links with many other determinants of health.^36^ In this study, marital status, history of spine/joint problems and behavioural factors accounted for 10–20% of the socioeconomic gaps in the PF decline rate. However, we were not able to further explore the influences of other time-varying covariates on the relationship between SEP and PF trajectories. In addition, the relationships of educational attainment and material condition with PF trajectories attenuated when both indicators were mutually adjusted for, but the patterns remained unchanged. This suggests both shared and independent effects of educational attainment and material condition on PF. More research is needed to advance our understanding of different indicators with PF trajectories, and how much other determinants of PF (eg, environmental factors, lifestyle, psychosocial factors, chronic conditions and injuries)^36^ mediate the socioeconomic inequalities observed in PF trajectories.

The relationship between education and change in self-rated health has been shown to be modified by birth cohort.^10^ ^11^ We did not to find such an effect on the PF decline rate. A similar lack of birth cohort effect was reported in previous studies examining wealth and trajectories of walking speed,^12^ and education and income with trajectories of physical impairment.^13^

An interesting finding of our investigation was the discrepancies in social differences in PF trajectories across the three countries. Some of these discrepancies may be due to random error. Alternatively, we can speculate that they may reflect a genuine lack of differences in access and accumulation of opportunities and resources between people with secondary and tertiary education in the Czech Republic and between those with less than secondary education and secondary education in Russia. The Czech cohort had a much larger proportion of participants with vocational education, reflecting not just historical trends^39^ but also the fact that while the Russian and Polish cohorts were selected from major cities with a population of over 1 million, the Czech cohort was based in seven middle-sized and smaller towns (population between 40 000 and 100 000). Given the relatively small difference in income between skilled manual workers and tertiary educated people,^39^ socioeconomic differences in access to resources related to good health may also be relatively small. In Russia, in contrast, vocational education was unpopular during the Soviet time.^39^ This speculation also seems supported by the differences in household amenities and assets by educational attainment in the three cohorts. There was a clear gradient of household amenities and assets across educational groups only in the Polish cohort, while material condition was similar between the upper two educational groups in the Czechs and between the lower two educational groups in the Russians.

The relatively slow PF decline with age in the Czech cohort may mirror a relatively better health status of the Czechs compared to the Russians and Poles, as exemplified by the higher life expectancy in the Czech Republic than in the other two countries.^33^ ^40^ ^41^ In the HAPIEE study, the Czech participants also reported better self-rated health, less prevalent cardiovascular disease, less depressive symptoms, less spine/joint problems, and more frequent contact with friends and relatives at baseline, compared with the Russians and Poles. Moreover, the subjective PF can be affected by medication, rehabilitation, change of environment (eg, removal of barriers) and use of assistive technology,^42^ and differential access to these interventions may also contribute to the cross-country differences in PF decline. Finally, the PF decline in the Czech cohort may not be large enough to allow detection of relatively minor disparities in PF trajectories across tertiles of material condition, or between the upper two educational groups. Consequently, a longer follow-up time may be needed to demonstrate socioeconomic differentials in PF in the Czech cohort.

## Conclusions

Educational attainment and material condition were positively associated with baseline PF status and the rate of PF decline over a 10-year follow-up period in middle-age and older Czechs, Russians and Poles. The widening socioeconomic disparities in PF trajectories were observed at all ages, supporting the CAD hypothesis. Marital status, spine/joint problems and behavioural factors accounted for around 10–20% of the socioeconomic differentials in the decline rate. A longer follow-up time and repeated measurements of SEP, PF and potential mediators are needed to further investigate the mechanisms.

**What is already known on this subject**

- Socioeconomic position is a well-established determinant of health associated with age-related decline in physical functioning. Two competing hypotheses have been proposed to explain the contradictory findings on socioeconomic differences in health over the lifespan: the cumulative advantage/disadvantage hypothesis predicts widening socioeconomic disparities in health with increasing age due to an accumulation of disadvantages over the life course, while the age-as-leveller hypothesis suggests diminishing socioeconomic gaps in health in late life because of the biological ceiling or selective mortality.

**What this study adds**

- We tested the cumulative advantage/disadvantage versus age-as-leveller hypothesis by examining the socioeconomic disparities in longitudinal changes of physical functioning with age in ageing populations in Central and Eastern Europe. In this investigation of three large ageing cohorts in the Czech Republic, Russia and Poland, higher educational attainment and ownership of more household amenities and assets were consistently associated with both better physical functioning at baseline and a slower rate of decline in physical functioning over the approximately 10 years of follow-up. The widening social disparities in physical functioning over time were found across the whole age range studied and in all cohorts, supporting the cumulative disadvantage hypothesis.
