# Supplementary material for: Education, material condition and physical functioning trajectories in middle-aged and older adults in Central and Eastern Europe: a cross-country comparison
Source: J Epidemiol Community Health. 2016 May 18;70(11):1128–35. doi: 10.1136/jech-2015-206548 (PMC5541176; doi:10.1136/jech-2015-206548)
Supplement: Supplementary data [file jech-2015-206548supp.pdf]

Supplementary tables

Supplementary table 1. Observed sample characteristics

	**Czech Republic**	**Russia**	**Poland**
Total	8773	9301	10709
Age (years, %)			
45.0-49.9	16.9	17.0	18.5
50.0-54.9	19.8	19.5	20.7
55.0-59.9	19.1	21.6	21.0
60.0-64.9	23.0	19.0	19.9
65.0-69.9	21.2	22.9	19.9
Missing (%)	0	0	0
Sex (%)			
Men	46.4	45.6	48.7
Women	53.6	54.4	51.3
Missing (%)	0	0	0
PF-10 score			
Baseline			
Mean	83.5	81.8	80.4
SD	18.8	20.4	21.4
Missing (%)	1.6	0	0.70
Re-examination			
Mean	83.1	80.3	74.6
SD	16.7	21.9	20.8
Missing (%)	41.2	33.9	38.3
PQ2009			
Mean	81.4	70.1	72.1
SD	20.5	26.9	26.4
Missing (%)	40.8	30.7	32.8
PQ2012			
Mean	81.2	67.3	68.2
SD	21.0	27.8	26.4
Missing (%)	47.6	50.4	65.6
Educational attainment (%)			
<Secondary education	49.5	36.9	32.7
Secondary education	36.3	34.2	38.7
University	13.7	28.9	28.5
Missing	0.5	0	0.10
Material conditions			
Mean	6.9	5.7	6.4
SD	2.3	2.1	2.2
%			
1st tertile	40.6	49.0	34.8
2nd tertile	29.7	29.2	45.4
3rd tertile	23.6	21.3	18.2
Missing (%)	6.1	0.5	1.6
Marital status (%)			
Married/cohabiting	75.4	72.4	76.1

Single/divorced/widowed	24.2	27.6	23.7
Missing	0.4	0	0.2
Spine/joint problems (%)			
No	42.8	34.6	30.5
Yes, never hospitalised	41.9	56.0	60.9
Yes, hospitalised	12.7	9.4	8.4
Missing	2.6	0	0.4
BMI (%)			
<25.0	20.0	27.5	22.0
25.0-29.9	36.7	37.5	37.7
≥30.0	25.3	35.1	26.7
Missing	18.0	<0.1	13.5
Drinking pattern (%)			
Non-drinking	12.4	15.8	34.3
Irregular light-to-moderate	30.6	42.8	31.4
Regular light-to-moderate	19.0	10.3	14.7
Irregular heavy	25.5	21.4	15.7
Regular heavy	9.0	9.7	3.4
Missing	3.5	<0.1	0.6
Smoking (%)			
Never	43.3	58.1	39.5
Former	29.1	13.6	28.2
Current	26.3	28.2	32.0
Missing	1.3	0	0.3

SD: standard deviation

Supplementary table 2. Initial status at baseline and slope of decline (per 1 year) in the PF-10 scores by educational attainment and material condition, stratified by country

	**Initial status**		**Slope**	
	Coefficient (95% CI)	p value	Coefficient (95% CI)	p value
**Czech Republic**				
Mean§	83.36 (81.96 to 84.76)	<0.01	-1.22 (-1.50 to -0.94)	<0.01
Variance§	189.77 (171.61 to 207.92)	<0.01	0.48 (0 to 0.96)	0.05
Baseline age (year)†	-0.33 (-0.38 to -0.28)	<0.01	-0.04 (-0.05 to -0.03)	<0.01
Female	-1.88 (-2.64 to -1.12)	<0.01	0.13 (-0.01 to 0.26)	0.06
Education				
<Secondary education	Reference		Reference	
Secondary education	2.16 (1.21 to 3.10)	<0.01	0.17 (0.03 to 0.31)	0.02
University	2.80 (1.85 to 3.74)	<0.01	0.22 (0.06 to 0.38)	<0.01
Material conditions				
1st tertile	Reference		Reference	
2nd tertile	2.52 (1.65 to 3.39)	<0.01	0.03 (-0.12 to 0.17)	0.71
3rd tertile	3.94 (3.04 to 4.83)	<0.01	-0.01 (-0.17 to 0.15)	0.89
**Russia**				
Mean§	84.88 (83.18 to 86.59)	<0.01	-2.96 (-3.39 to -2.54)	<0.01
Variance§	134.31 (111.26 to 157.35)	<0.01	1.16 (0.38 to 1.95)	<0.01
Baseline age (year)†	-0.46 (-0.52 to -0.40)	<0.01	-0.10 (-0.12 to -0.09)	<0.01
Female	-6.51 (-7.54 to -5.48)	<0.01	-0.04 (-0.28 to 0.21)	0.78
Education				
<Secondary education	Reference		Reference	
Secondary education	-0.46 (-1.38 to 0.47)	0.33	0.04 (-0.14 to 0.23)	0.66
University	2.09 (1.18 to 3.00)	<0.01	0.44 (0.25 to 0.62)	<0.01
Material conditions				
1st tertile	Reference		Reference	
2nd tertile	4.16 (3.27 to 5.05)	<0.01	0.12 (-0.06 to 0.30)	0.20
3rd tertile	4.86 (3.86 to 5.86)	<0.01	0.36 (0.14 to 0.57)	<0.01
**Poland**				
Mean§	82.48 (80.92 to 84.04)	<0.01	-2.40 (-2.72 to -2.08)	<0.01
Variance§	190.41 (165.23 to 215.60)	<0.01	0.98 (0.50 to 1.46)	<0.01
Baseline age (year)†	-0.53 (-0.59 to -0.47)	<0.01	-0.05 (-0.06 to -0.04)	<0.01
Female	-4.35 (-5.17 to -3.52)	<0.01	-0.15 (-0.31 to 0.01)	0.07
Education				
<Secondary education	Reference		Reference	
Secondary education	0.91 (-0.03 to 1.85)	0.06	0.32 (0.14 to 0.50)	<0.01
University	2.83 (1.86 to 3.80)	<0.01	0.58 (0.38 to 0.78)	<0.01
Material conditions				
1st tertile	Reference		Reference	
2nd tertile	3.53 (2.56 to 4.50)	<0.01	0.11 (-0.06 to 0.28)	0.22
3rd tertile	4.92 (3.84 to 6.00)	<0.01	0.25 (0.04 to 0.45)	0.02

* p<0.05, ** p<0.01, *** p<0.001; § conditional on the covariates adjusted in the model; † centred on 58 years
Adjusted for baseline age, sex, marital status, history of spine/joint problems, BMI, drinking pattern and smoking status

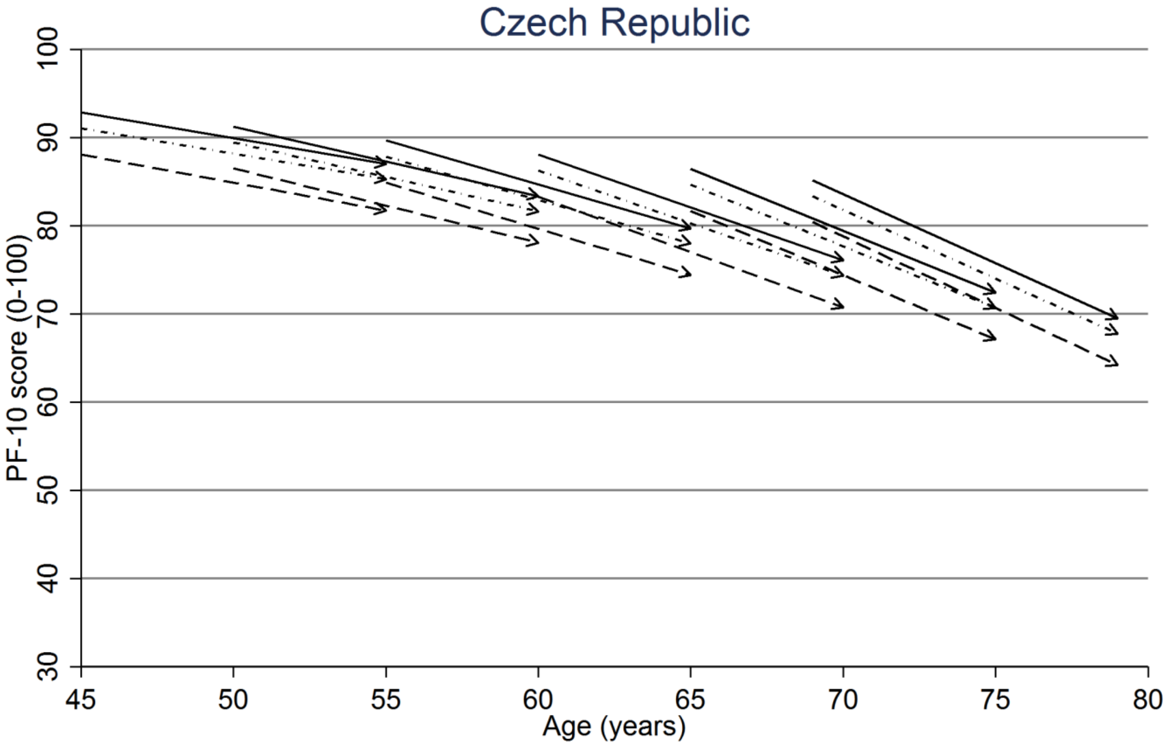

Czech Republic
PF-10 score (0-100)
100
90
80
70
60
50
40
30
45
50
55
60
65
70
75
80
Age (years)

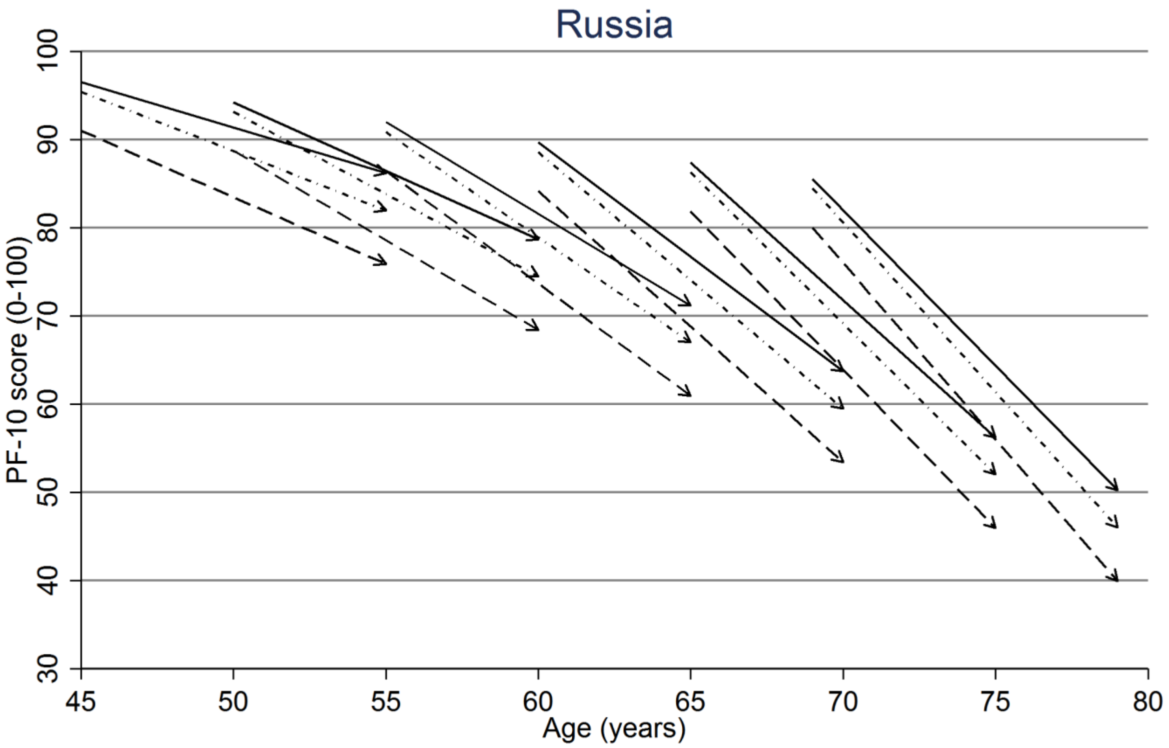

Russia
PF-10 score (0-100)
100
90
80
70
60
50
40
30
45
50
55
60
65
70
75
80
Age (years)

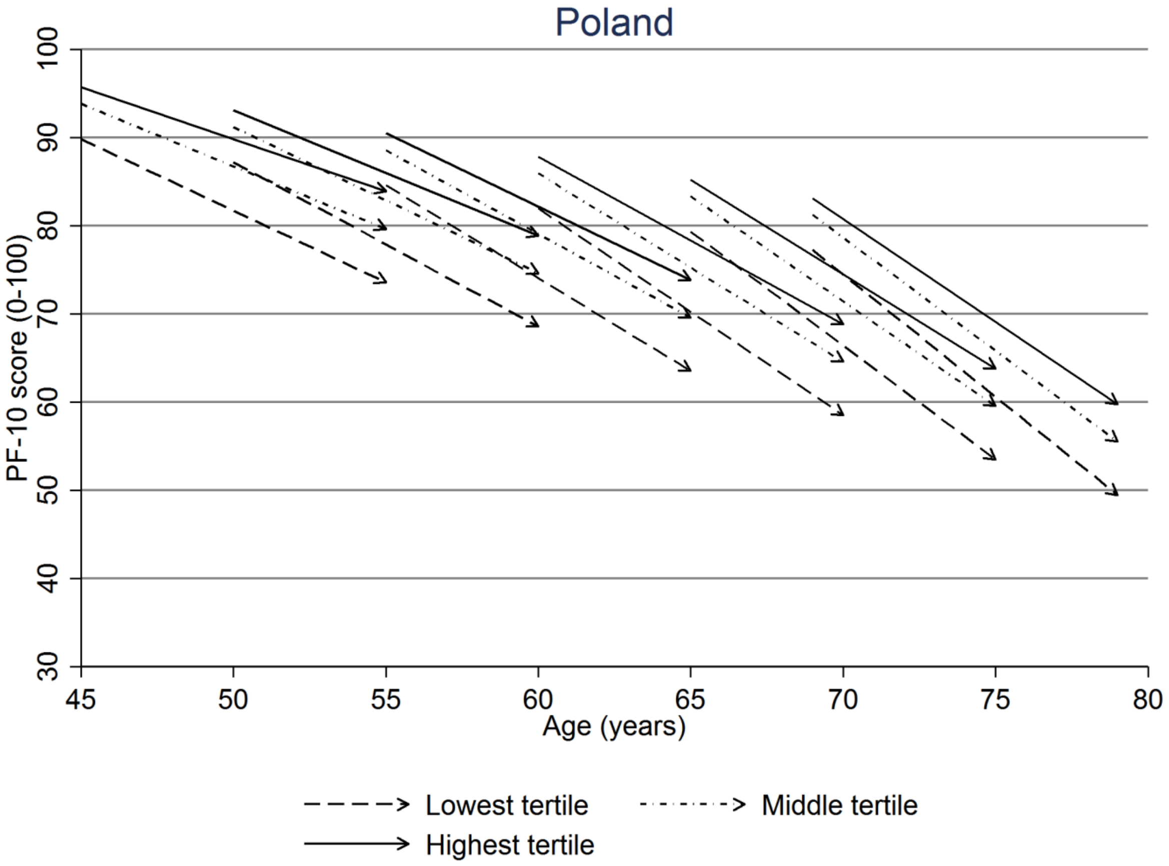

Supplementary figure 1. Vector graphs of predicted initial status and slope of PF-10 score during 10-year follow-up for every fifth one-year birth cohort by material conditions (model 2)
